# Paraneoplastic syndrome as the presentation of limited stage small cell carcinoma

**DOI:** 10.1186/s12890-018-0729-y

**Published:** 2018-11-14

**Authors:** Kia Nikoomanesh, Julian Choi, Sarkis Arabian

**Affiliations:** 1 Department of Internal Medicine, Arrowhead Regional Medical Center, Colton, California USA; 2 Department of Pulmonary and Critical Care Medicine, Arrowhead Regional Medical Center, Colton, California USA

**Keywords:** Small cell lung carcinoma, Paraneoplastic syndrome, Syndrome of inappropriate ADH secretion, Hyponatremia

## Abstract

**Background:**

Small cell lung carcinoma (SCLC) is one of the deadliest forms of lung cancer due to its poor prognosis upon diagnosis, rapid doubling time, and affinity for metastasis. As 60–70% of patients with SCLC have disseminated disease upon presentation, it is imperative to determine the extent of disease burden for treatment. As a neuroendocrine carcinoma, clinicians must pay close attention to abnormal findings in a smoker that could lead to earlier diagnosis and better prognostication.

**Case presentation:**

A 64 year-old 20-pack year smoker presented to the emergency department with nausea and vomiting for 3 days. No inciting events were elicited. History and review of symptoms were negative including symptoms most-commonly associated with malignancy such as fevers and weight loss. He also denied any pulmonary symptoms. Physical examination was benign except for right lung end-expiratory wheezing. Our patient was clinically euvolemic. Initial blood laboratories showed a sodium 110, serum osmolarity 227, and urine osmolarity of 579. Fluid restriction led to normalization of his sodium and resolution of nausea & vomiting. Chest radiography was obtained to follow-up on the wheezing, which was read as no acute cardiopulmonary disease by radiology. Due to high suspicion of SIADH from malignancy, a CT of the chest was performed which showed a conglomerate of nodules and opacities in the right upper lobe. Biopsy revealed SCLC. At outpatient follow-up, patient had a PET-CT showing one active mediastinal lymph node as the only site of metastasis. He received three round of chemotherapy, chest and prophylactic cranial radiation, and deemed in remission by oncology.

**Discussion and conclusions:**

Due to its affinity for metastases, 70% of patients with SCLC present with symptoms related to the spread of cancer to affected organ systems. Given the aggressive nature of this disease, screening measures have been implemented to help diagnose limited stage SCLC. Unfortunately, in our patient and many others, screening guidelines may fail to identify appropriate patients to scan. It is therefore imperative to use our clinical index of suspicion and identify any early presentations (including paraneoplastic syndromes) which may tip off the beginning stages of SCLC. This could improve survival rates by up to 45%.

## Background

Small cell lung cancer (SCLC) is a highly aggressive form of pulmonary malignancy which accounts for 15–20% of lung cancer cases [1]. SCLC is well known for its rapid doubling time, tendency to metastasize, and its affinity for relapsing. Nearly 60–70% of patients with SCLC present with disseminated disease [1], and early diagnosis improves outcomes. Typical presentations include cough, dyspnea, and weight loss. However, SCLC can present in more atypical ways.

Paraneoplastic syndromes such as syndrome of inappropriate antidiuretic hormone secretion (SIADH), Lambert-Eaton myasthenic syndrome, hypercalcemia, and Cushing’s syndrome are some atypical presentations. Up to 10% of patients with lung cancer develop a paraneoplastic syndrome during the course of their disease progression [2]. An even smaller number of patients have paraneoplastic syndromes as their presenting feature. It is therefore not surprising that abnormal laboratory values can be misinterpreted as isolated findings when there is no obvious evidence of malignancy.

SCLC has almost an exclusive association with cigarette smoking. In one study, smoking was associated in 100 and 97.9% of cases in men and women, respectively [3]. With this strong of a relationship, SCLC should be considered in current and past smokers with evidence of abnormal laboratories or symptoms consistent with paraneoplastic syndromes. The following case depicts how identifying a paraneoplastic syndrome helped uncover an underlying SCLC and improve one patient’s prognosis.

## Case discussion

A 64 year-old caucasian male presented with a chief complaint of nausea and vomiting. These episodes occurred three-to-four times per day for the past 3 days and were non-bilious, non-bloody, mostly foodstuff. There were no precipitating factors or associated symptoms including abdominal pain or diarrhea. He did not complain of any recent pulmonary symptoms such as cough, hemoptysis, dyspnea, or chest pain, and denied any fevers, night sweats, or weight loss. He had no past medical history except for hypercholesterolemia controlled with atorvastatin. His only family history included Hodgkin’s lymphoma. Smoking history revealed 20 pack years and quit 3 months prior to his visit. Vital signs upon presentation were unremarkable. Physical examination revealed mild right upper lung field end-expiratory wheezing, no clubbing of his digits, no jugular venous distention, no lower extremity edema, was euvolemic, and had unremarkable abdominal findings.

**Fig. 1:**
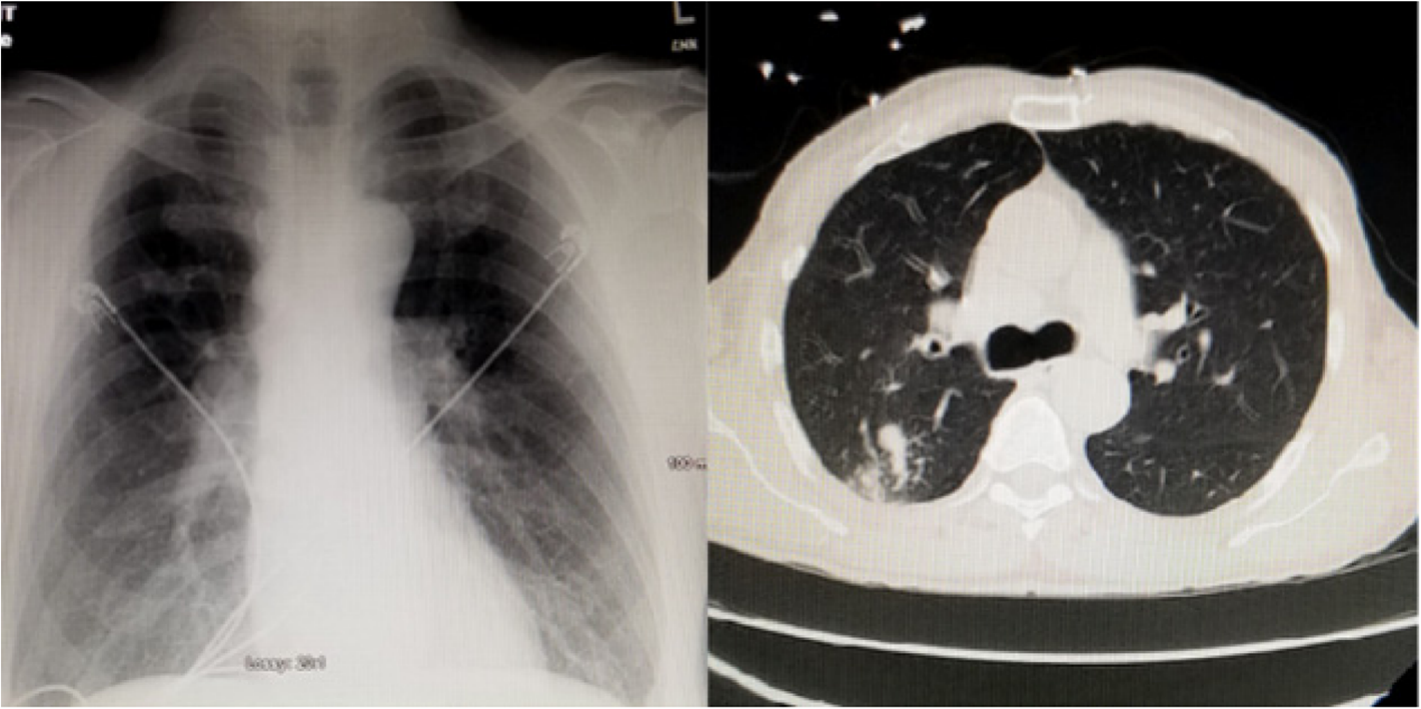
Left: AP chest plain film read as no acute cardiopulmonary disease. Right: CT chest showing a conglomeration of nodules and opacities measure 3.0 × 1.9 cm of the inferior portion of the right upper lobe

An anterior-posterior chest plain film (Fig. 1) was performed in the emergency department, and read by the radiologist as having no evidence of acute cardiopulmonary disease. Laboratories drawn on admission revealed hyponatremia in the context of a low serum osmolality and a high urine osmolality (Table 1). Given these laboratory findings, SIADH ranked high in our differential diagnoses.

**Table 1:** Blood laboratories from day 1, 2, and 6

Laboratory Studies	Day 1	Day 2	Day 6
Sodium (mEq/L)	110	119	130
Blood Urea Nitrogen (mg/dL)	7	9	16
Creatinine (mg/dL)	0.5	0.5	0.6
Serum osmolality (mOsm/kg)	227	237	
Urine osmolality (mOsm/kg)	579		
Urine sodium (mEq/L)	126		
Adrenocorticotropic hormone (pg/mL)	15		
Total cortisol (ug/dL)	16.55		
Thyroid stimulating hormone (mIU/mL)	1.59		

Treatment with fluid restriction was initiated and sodium levels gradually improved (Table 1). The patient’s nausea and vomiting had resolved as his sodium levels improved, which later was attributed to his hyponatremia from SIADH. Potential etiologies for SIADH (i.e. infectious, cerebral, medications, endocrinopathies) were further investigated and were unremarkable. Due to the patient’s significant smoking history, unilateral end-expiratory wheeze, initial poor quality chest imaging, and high-index of suspicion, a CT chest was ordered. It revealed the presence of a conglomeration of nodules and opacities measuring 3.0 × 1.9 cm in the inferior segment of the right upper lobe of the lung with ipsilateral mediastinal lymphadenopathy. Subsequent CT-guided percutaneous biopsy was performed. Biopsy specimen stained positive for CK, CD56, NSE, BcL2, Synaptophysin, PAX-5, and TTF-1, but negative for Chromogranin, CD57, and NFP; consistent with small cell carcinoma. Further metastatic workup including CT abdomen and pelvis along with brain MRI was performed, which showed no evidence of metastasis.

He was subsequently discharged with an appointment with an outside oncologist near his home as he lived far from our institution. A follow-up telephone conversation occurred 4 weeks from discharge. The patient’s oncologist had ordered a whole-body PET scan within 1 week of hospital discharge and it revealed uptake in one ipsilateral mediastinal lymph node. He was classified as having limited stage small cell lung cancer (LS-SCLC) and was undergoing chemotherapy with plans for subsequent prophylactic cranial irradiation. At 4 months follow-up, he had completed three chemotherapy sessions and prophylactic cranial irradiation. His oncologist had deemed him to be in remission and undergoing continued surveillance.

## Discussion and conclusions

SCLC can present with three possible manifestations: pulmonary, metastatic, and endocrinologic/neurologic paraneoplastic syndromes. The most common of these three manifestations is metastatic as approximately 70% of patients with SCLC present with metastatic disease [4]. This is a clear testament to the highly aggressive nature of this disease process. For this reason, screening measures have been implemented to help identify SCLC at an earlier stage. Current US Preventive Services Task Force (USPSTF) guidelines recommend screening individuals between 55 and 80 years old with at least a 30 pack year smoking history for lung cancer. The screening modality recommended is a low dose CT scan of the chest. This guideline has been supported by trials such as the national lung screening trial (NLST) which showed a 20% reduction in mortality in patients who met criteria and were screened appropriately.

Unfortunately, as seen in our patient, screening based on age and pack year history alone can lead to missed diagnoses.More recent studies suggest that a screening model using individual risk factors could be superior to the current established guideline. One study investigated the efficacy of nine different screening models based on various risk factors [5]. Risk factors in these screening models included: gender, race, ethnicity, education, body mass index, chronic obstructive pulmonary disease, emphysema, personal history of cancer, personal history of pneumonia, and family history of lung cancer. Results from this study suggest increased sensitivity and specificity for risk factor based screening groups in comparison to NLST guideline based groups. However, both the USPSTF guidelines, and risk factor based screening models have the potential to miss early diagnoses. Our case outlines a patient who does not fit the USPSTF guidelines for screening and does not fit perfectly into any risk factor based screening model.

Therefore, it continues to be a diagnostic challenge to detect early stage lung cancer such as SCLC. The aggressive nature of SCLC leaves physicians with a narrow window for discovering LS-SCLC. Despite advances in screening guidelines and modalities patients often fail to meet criteria and therefore go unnoticed before they present with metastatic disease. Given the possible shortcomings of screening modalities, physicians must rely on their clinical index of suspicion to help discover early disease. This includes a holistic approach to each patient, correlating possible early symptoms of presentation with abnormal lab values that could indicate a possible paraneoplastic syndrome. Identifying these underlying paraneoplastic syndromes may be the key to discovering a LS-SCLC in a patient who otherwise appears asymptomatic. This could significantly alter a patient’s disease course and improve a 5 year survival rate by nearly 45% [5].

## Abbreviations

BUN/Cr: Blood urea nitrogen/creatinine ratio
ES-SCLC: Extensive-stage small-cell lung carcinoma
LS-SCLC: Limited-stage small-cell lung carcinoma
mEq/L: Milli-equivalents per liter
mg/dL: Milligram per deciliter
mg/L: Milligram per liter
mIU/mL: milli-international units per milliliter
mOsm/kg: milliosmoles per kilogram
pg/mL: Nanogram per milliliter
SCLC: Small cell lung carcinoma
SIADH: Syndrome of inappropriate antidiuretic hormone secretion
ug/dL: Microgram per deciliter
USPSTF: US Preventive Services Task Force
